# Age‐Associated Expansion of HIV/SIV Reservoirs in People With HIV and SIV‐Infected Macaques

**DOI:** 10.1111/acel.70252

**Published:** 2025-10-06

**Authors:** Arpan Acharya, Debapriya Sutar, Mahesh Mohan, Varan Govind, Francois Villinger, Suresh Pallikkuth, Siddappa N. Byrareddy

**Affiliations:** ^1^ Department of Pharmacology and Experimental Neuroscience University of Nebraska Medical Center Omaha Nebraska USA; ^2^ Southwest National Primate Research Center Texas Biomedical Research Institute San Antonio Texas USA; ^3^ Department of Radiology University of Miami Miller School of Medicine Miami Florida USA; ^4^ Department of Biology University of Louisiana at Lafayette Lafayette Louisiana USA; ^5^ Department of Microbiology and Immunology University of Miami Miller School of Medicine Miami Florida USA

**Keywords:** antiretroviral therapy (ART), CD4+ T cells, human immunodeficiency virus‐1 (HIV), intact proviral DNA, people living with HIV (PWH), reservoirs, simian immunodeficiency virus (SIV)

## Abstract

With the advancement of combination antiretroviral therapy (ART), over 50% of people with HIV (PWH) in the United States are now over the age of 50. A hallmark of the aging immune system is a progressive dysfunction of both the innate and adaptive immune responses, often characterized by clonal expansion of memory T cells. However, the impact of age‐related immune dysfunction on HIV/SIV reservoir dynamics remains understudied. We hypothesized that age‐associated clonal expansion of memory T cells contributes to the increase of the HIV reservoirs in older PWH (OPWH). In this retrospective study, we utilized archived peripheral blood mononuclear cells (PBMCs) from young and older PWH with suppressed plasma viremia for at least 5 years and quantified both intact and total HIV proviral DNA from CD4+ T cells. Alongside the human study, we also analyzed samples from SIV‐infected, ART‐suppressed young and aged rhesus macaques, quantifying intact and total proviruses in CD4+ T cells. We observed a significantly higher level of intact and total proviral DNA in older compared to younger PWH. The frequency of intact provirus was positively correlated with activated CD4+ and CD8+ T cells. Consistently, in the non‐human primate model, aged macaques exhibited significantly higher levels of intact and total SIV proviruses in CD4+ T cells than their younger counterparts. Collectively, these findings suggest that the HIV/SIV reservoir expands with age, potentially driven by immune activation. Future studies are warranted to elucidate the mechanisms underlying reservoir expansion in the aging population.

## Graphical abstract

Virally suppressed older people with HIV have larger peripheral blood CD4+ T‐cell reservoirs than younger individuals, correlating with CD4+ and CD8+ T‐cell activation. Similar results were seen in SIV‐infected, ART‐treated aged rhesus macaques.

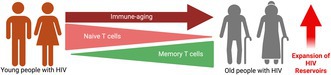

## 1 Introduction

The efficacy of combination antiretroviral therapy (ART) has extended the life expectancy of people living with HIV (PWH) (Trickey et al. 2023). In the United States, over 50% of PWH are now 50 years or older, and by 2030, this number is expected to exceed 70% (Wing 2016). Notably, ART does not prevent cell‐to‐cell transmission of HIV (Lan et al. 2022), which limits the decay rate of latent reservoirs. Aged individuals naturally experience progressive immune dysfunction, characterized by chronic inflammation and alteration in CD4/CD8 ratios (Panda et al. 2009; Ponnappan and Ponnappan 2011; Weng 2006). Several factors contribute to this process, including thymic involution (Calder et al. 2011); mitochondrial dysfunction leading to a proinflammatory phenotype in T cells (Quinn et al. 2019; Xu et al. 2021; Younes et al. 2018); epigenetic alterations in T cells; and naïve‐memory T‐cell imbalance Clonal expansion of effector memory T cells with simultaneous loss of naïve T cells (Goronzy et al. 2015) and latent reservoirs of HIV persist by the clonal expansion of effector memory T cells (Yeh et al. 2021).

Aging is associated with the accumulation of terminally differentiated T cells that acquire a senescent/exhausted phenotype, and HIV reservoirs are enriched in PD1, TIM‐3, and TIGIT‐expressing exhausted CD4+ T cells (Fromentin et al. 2019). With aging, T cells also lose their functional plasticity and have a biased lineage commitment toward Th1 and Th17 cells associated with the secretion of elevated levels of circulating inflammatory cytokines like IL‐6, which contribute to inflammation (Elyahu et al. 2019). Therefore, T‐cell aging is expected to influence the size of the HIV reservoirs in older PWH (OPWH) compared to their younger counterparts. However, to the best of our knowledge, the current literature lacks studies that directly compare HIV reservoir sizes between younger and older PWH, a gap that holds significant implications for cure‐focused research. In this pilot study, we compared the HIV reservoir sizes in younger and older PWH and supported these findings with data from a parallel study involving SIV‐infected, ART‐treated young and aged rhesus macaques (RMs).

## 2 Results and Discussion

**FIGURE 1:**
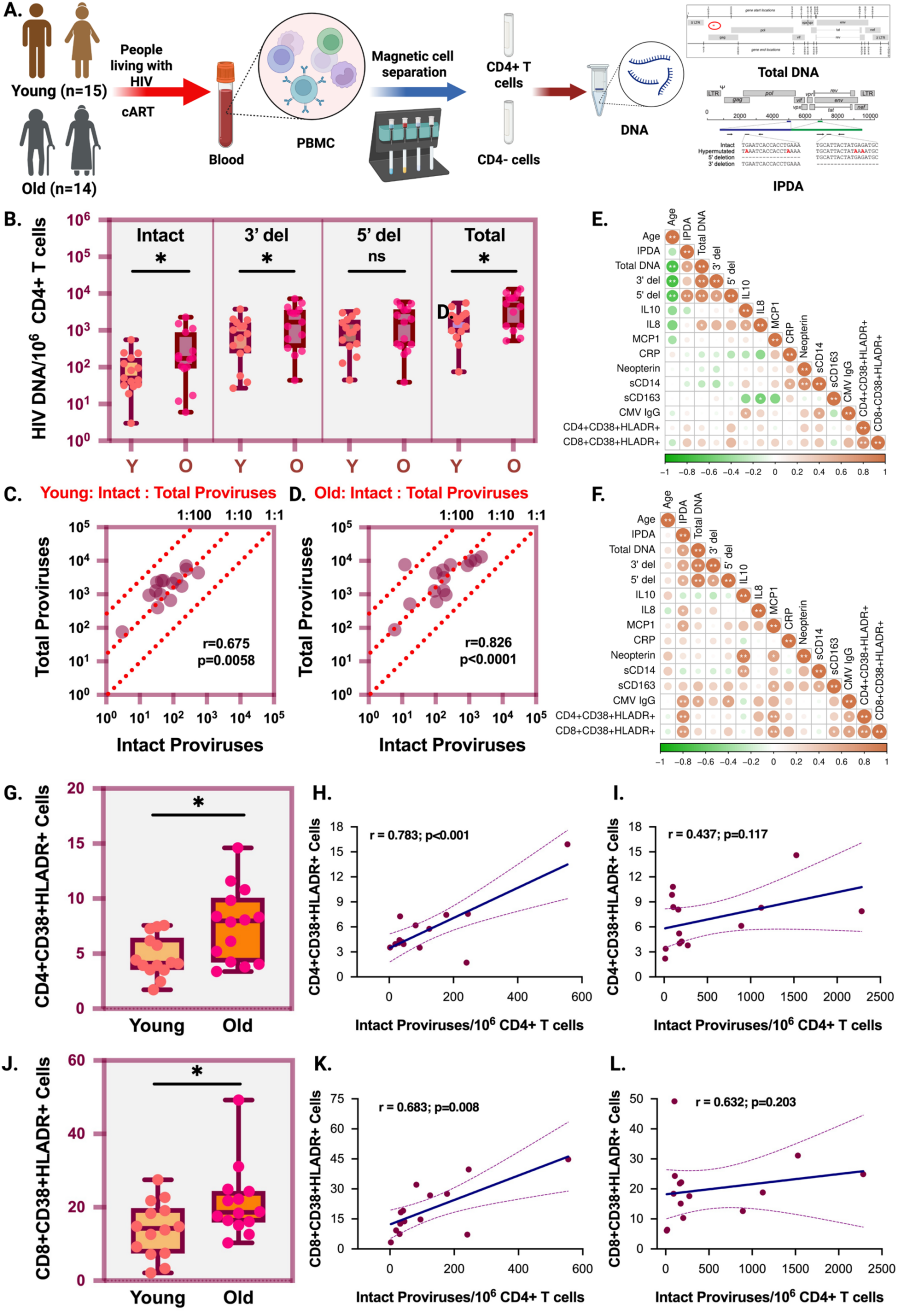
Comparison of HIV reservoir levels in peripheral blood from young and old people living with HIV (PWH). (A) The experimental outline of measuring total and intact proviral DNA in CD4+ T cells purified from PBMC of PWH; (B) Comparison of intact, 3′ deleted, 5′ deleted, and total proviral DNA per million CD4+ T cells in PBMCs between young versus old PWH; (C) Spearman correlation plot between intact and total HIV proviral DNA levels in CD4+ T cells from PBMCs of young PWH; (D) Spearman correlation plot between intact and total HIV proviral DNA levels in CD4+ T cells from PBMCs of old PWH; The dashed lines correspond to relative ratios of intact to total proviral DNA; (E) Spearman correlation plot of intact and total proviral DNA in CD4+ T cells from PBMCs, plasma IL‐10, IL‐8, MCP1, CRP, neopterin, cCD14, sCD163, CMV IgG %CD4+CD38+HLADR+, and %CD8+CD38+HLADR+ cells in PBMCs from the young PWH; (F) Spearman correlation plot of intact and total proviral DNA in CD4+ T cells from PBMC, plasma IL‐10, IL‐8, MCP1, CRP, neopterin, cCD14, CD163, CMV IgG, %CD4+CD38+HLADR+, and %CD8+CD38+HLADR+ cells in PBMC from the old PWH; (G) Comparison of the percentage of CD4+CD38+HLADR+ T cells among young and old PWH; (H) Spearman correlation plot between intact HIV proviral DNA levels and percentage of CD4+CD38+HLADR+ T cells in PBMC from young PWH; (I) Spearman correlation plot between intact HIV proviral DNA levels and percentage of CD4+CD38+HLADR+ T cells in PBMC from old PWH; (J) Comparison of the percentage of CD8+CD38+HLADR+ T cells among young and old PWH; (K) Spearman correlation plot between intact HIV proviral DNA levels and percentage of CD8+CD38+HLADR+ T cells in PBMCs from young PWH; (L) Spearman correlation plot between intact HIV proviral DNA levels and percentage of CD8+CD38+HLADR+ T cells in PBMCs from old PWH; Young PWH are aged 40 years and below and old PWH are aged 60 years and above at the time of study entry; ** indicates *p* < 0.005, * indicates *p* < 0.05 and ns: not significant; In the correlation plots, the positive and negative correlation values are indicated by the color bar and the circle size.

Using archived samples, we measured the HIV reservoirs in PBMCs of PWH using the Intact Proviral DNA Assay (IPDA) (Bruner et al. 2019). The details of our study cohort are described in Table S1 (IRB # 20170687). The young cohort is defined as PWH under 40 years of age, and the older cohort is defined as PWH 60 years and above. The mean age of young PWH (YPWH; *n* = 15) was 28.7 years, while the mean age of OPWH (*n* = 14) was 64.6 years at the time of study entry. All the study participants were on ART for more than 5 years and had plasma viral loads below 20 copies/mL at the time of sample collection. The experimental outline of the study is described in Figure 1A. In brief, CD4+ T cells were purified from PBMCs using the EasySep Human CD4+ T‐Cell Isolation Kit (StemCell Technologies, Canada; Cat#100‐0696). We also collected the fraction of PBMCs negative for CD4+ T cells, hereafter referred to as CD4− cells, from the samples. Genomic DNA from CD4+/CD4− cells was isolated using the QIAamp DNA Mini Kit (Qiagen; Germany; Cat# 51304), and the number of intact and total HIV proviral DNA was measured using a Bio‐Rad QX200 AutoDG Digital Droplet PCR system as described previously (Bruner et al. 2019). When YPWH and OPWH were compared, a significantly higher level of intact (median value of 176 vs. 51) and total proviral DNA (median value of 4827 vs. 1932) copies per million CD4+ T cells was detected in OPWH (Figure 1B). Similarly, we observed a significantly higher level of 3′ defective proviruses among OPWH. In contrast, there were differences in 5′ defective proviruses between the groups that were not statistically significant. It is well established that the envelope region located at the 3′ end of the HIV genome is more prone to mutations than the 5′ end of the genome (Cuevas et al. 2015). This inherent genomic instability at the 3′ end may contribute to our observation of no significant differences in 5′ deleted proviruses between younger and older PWH. Peluso et al. used mathematical models to show that after ART begins, intact proviruses decay by 15.7% per year for the first 7 years, then slow to a rate of 3.6% per year. In contrast, the 3′ defective proviruses decay at the rate of 5.9% per year for the first 7 years, followed by a reduced rate of 0.9% per year. Interestingly, 5′ defective proviruses were found to be stable, with a 1.2% increase per year for the first 7 years of suppression, followed by a decline of 1.5% per year thereafter (Peluso et al. 2020). Mechanistically, it was proposed that cells harboring intact proviruses have a higher likelihood of producing viral proteins and are thus more effectively cleared by the host immune response compared to the defective proviruses (Ho et al. 2013; Imamichi et al. 2016). Moreover, the clonal expansion of long‐lived CD4+ T‐cell reservoirs at a later stage of viral suppression may contribute to the reduced decay rate of the viral reservoirs over time in PWH. Collectively, these factors may explain the lack of significant difference in the frequency of 5′ deleted proviruses between YPWH and OPWH in our study cohort. In contrast to the findings of Peluso et al., other studies have shown that in PWH on long‐term ART, after 7 years, viral reservoirs no longer decline but instead begin to expand, with an estimated doubling time of 23 years, suggesting the proliferation of infected cells (McMyn et al. 2023). There was a positive correlation between the intact and total HIV proviral DNA copies in both young and aged PWH as shown in Figure 1C,D. Furthermore, similar to previous reports, we noted substantial interindividual variability in the ratio of intact to total proviral DNA in both groups (Gandhi et al. 2021; Peluso et al. 2020). Next, we performed a correlation analysis between intact, total, 5′ defective, and 3′ defective proviruses, plasma cytokine/chemokines, CMV IgG, and activated CD4/CD8 T cells (CD38+HLADR+) in peripheral blood and found notable differences in correlation coefficients between the groups (Figure 1E,F). Notably, in YPWH, we observed a statistically significant positive correlation between the level of CMV IgG and the frequency of intact proviruses. Additionally, in OPWH, the frequency of CD4+CD38+HLADR+ T cells was significantly higher than those found in YPWH (Figure 1G), and a positive correlation was observed between the frequency of intact proviruses and %CD4+CD38+HLADR+ T cells in both groups (Figure 1H,I). Similarly, the frequency of the %CD8+CD38+HLADR+ T cells was significantly elevated in OPWH compared to the younger group (Figure 1J), with a corresponding positive correlation between the frequency of intact proviruses and %CD8+CD38+HLADR+ T cells in both groups (Figure 1K,L). Previous studies have shown that HIV reservoirs were enriched in CD38+CD4+ T_CM_ cells compared to CD38‐CD4+ T_CM_ cells in long‐term virally suppressed PWH (Song et al. 2020). During chronic infection under suppressive ART, HIV proviral DNA load was positively correlated with HLA‐DR+CD8+ T lymphocytes (Zhu et al. 2023). Additionally, in pediatric HIV, an elevated population of CD38+CD8+ T cells has been linked to increased mortality within the first year of life (Sherman et al. 2002). In lymph nodes, HLA‐DR+CD38+CD4+ T cells from PWH overexpressed CCR5 and produced most of the infectious virions (Meditz et al. 2011). Moreover, studies reported an expansion of CD38‐expressing terminally differentiated effector memory CD8+ T cells in OPWH with suppressed plasma viremia, along with an expansion of the senescence marker CD57 (Shin et al. 2022). Taken together, these findings suggest that the expansion of activated memory CD4+ and CD8+ T cells with a senescent phenotype may be driving the expansion of viral reservoirs in OPWH. However, the underlying mechanisms warrant further investigation in future studies.

Next, to understand the contribution of sex differences in HIV reservoirs, we compared the frequency of intact and total proviruses in CD4+ T cells between male and female PWH. When analyzing the young and old cohorts together, we found that the median values of total proviruses per million CD4+ T cells in males were higher than those in females, although the difference was not statistically significant (Figure S1A). Similarly, we did not observe a significant difference in intact proviruses between males and females (Figure S1B). These patterns remained consistent when comparing male and female data from the young and aged cohorts individually (Figure S1C–F). Several studies mirrored our findings of no significant difference in total HIV‐1 DNA levels in CD4+ T cells from PBMCs of men and women living with HIV on long‐term ART (Falcinelli et al. 2020; Gianella et al. 2020; Scully et al. 2019). Due to the limited number of available PBMCs, we were unable to purify myeloid cells. Instead, we performed IPDA on CD4− cells to estimate the myeloid reservoirs. In contrast to CD4+ T cells, we did not observe any significant differences in intact, 3′ defective, 5′ defective, and total proviruses in YPWH versus OPWH (Figure S2A). However, we observed a positive correlation between the intact and total HIV proviral DNA copies in both groups, with the correlation reaching statistical significance in OPWH (Figure S2B,C). In summary, we found a significantly higher level of intact and total proviruses in CD4+ T cells from PBMCs of OPWH compared to their younger counterparts, and the level of intact proviruses was positively correlated with CD38+HLADR+ CD4+ and CD8+ T cells.

**FIGURE 2:**
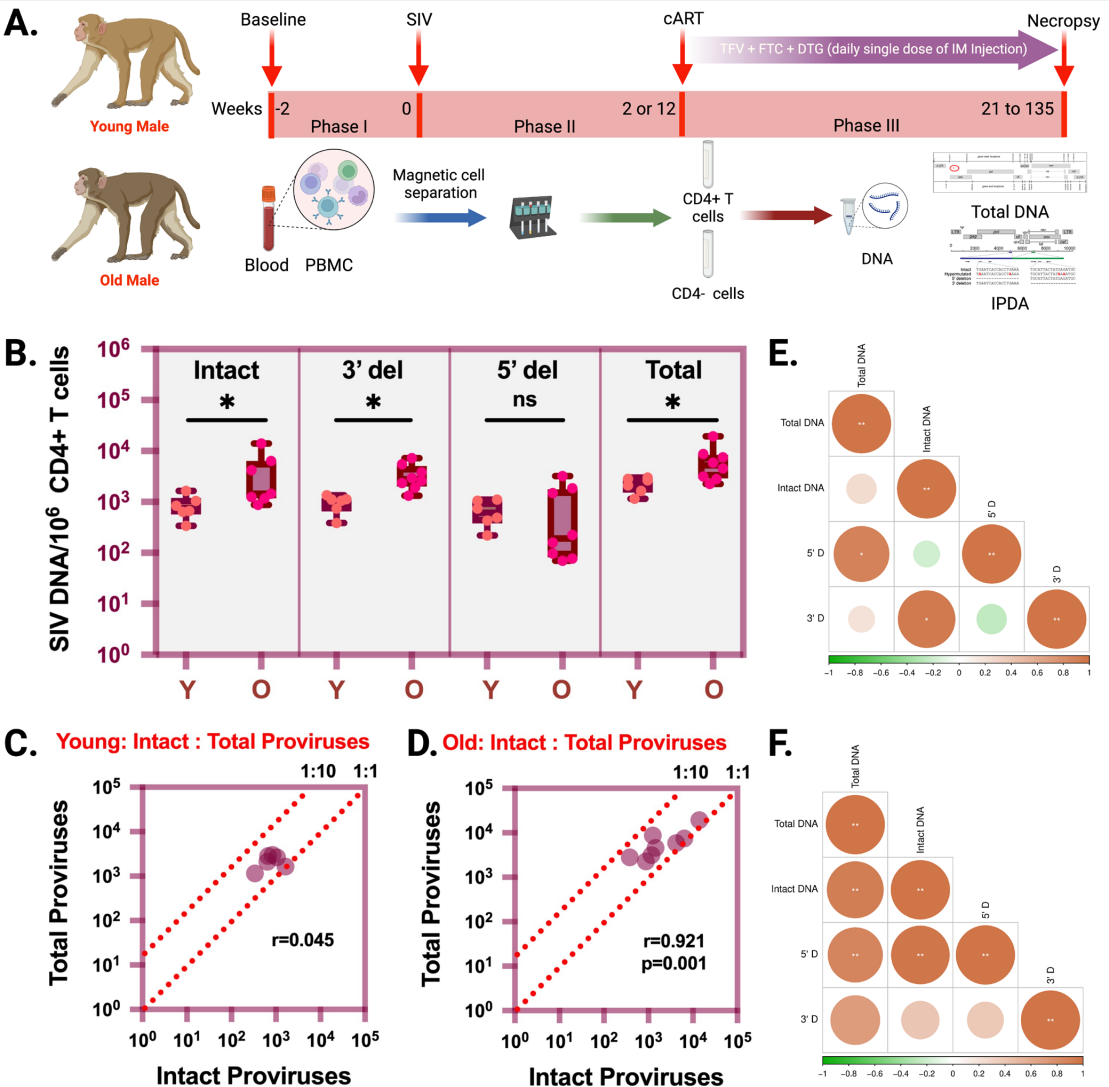
Comparison of SIV reservoir levels in peripheral blood from young and old rhesus macaques. (A) The experimental outline of measuring total and intact proviral DNA in CD4+ T cells purified from PBMCs of SIV‐infected ART‐treated rhesus macaques (RMs); (B) Comparison of intact, 3′ deleted, 5′ deleted, and total proviral DNA per million CD4+ T cells in PBMCs between young versus old SIV‐infected ART‐treated rhesus macaques; (C) Spearman correlation plot between intact and total SIV proviral DNA levels in CD4+ T cells from young SIV‐infected ART‐treated rhesus macaques; (D) Spearman correlation plot between intact and total SIV proviral DNA levels in CD4+ T cells from old SIV‐infected ART‐treated rhesus macaques; The dashed lines correspond to relative ratios of intact to total proviral DNA; (E) Spearman correlation plot of total, intact, 5′ deleted, and 3′ deleted SIV proviral DNA in CD4+ T cells obtained from PBMCs of young SIV‐infected ART‐treated RMs; (F) Spearman correlation plot of total, intact, 5′ deleted, and 3′ deleted SIV proviral DNA in CD4+ T cells obtained from PBMC of old SIV‐infected ART‐treated RMs; Young rhesus macaques were 2–5 years old and old rhesus macaques were above 19 years old at the time of study entry; ** indicates *p* < 0.005, * indicates *p* < 0.05 and ns: not significant; 5′ D: 5′ deleted and 3′ D: 3′ deleted; In the correlation plots, the positive and negative correlation values are indicated by the color bar and the circle size.

In a complementary study, we compared SIV reservoirs in young and aged male Indian‐origin rhesus macaques (RMs). Young (2–5‐year‐old male; *n* = 6) and aged (over 19 years old; three female and five male; a subset of these animals received THC/CBD adjuvant therapy to assess its neuroprotective capacity in the brain; once daily IM injection of 0.18 mg/kg delta‐9‐tetrahydrocannabinol (THC) and 0.54 mg/kg cannabidiol (CBD)) (Okeoma et al. 2024). RMs were infected with SIV and treated with combined antiretroviral therapy (ART: including TDF (5.1 mg/kg), FTC (40 mg/kg), and DTG (2.5 mg/kg) once daily by subcutaneous injection as 1 mL/kg body weight) (Acharya et al. 2020). After achieving complete suppression of plasma viremia for at least 6 months, CD4+ T cells were purified from PBMCs using the EasySep NHP CD4+ T‐cell isolation kit (STEMCELL Technologies Canada Inc., cat. no. 19582), and the intact and total SIV proviral DNA was quantified as described in Figure 2A (Acharya et al. 2020; Bender et al. 2019). The details of the rhesus macaque samples used in this study are provided in Table S2.

When compared between groups, aged RMs exhibited significantly higher levels of both intact (median: 1444 vs. 752) and total SIV proviral DNA (median: 5225 vs. 2379) per million CD4+ T cells from PBMC compared to young RMs. Similarly, the level of 3′ defective proviruses was significantly elevated in aged RMs compared to young macaques. In contrast, the differences in 5′ defective provirus levels between the groups were not statistically significant (Figure 2B). A positive correlation was observed between the frequencies of intact and total SIV proviral DNA copies per million CD4+ T cells in both groups, reaching statistical significance in aged RMs (Figure 2C,D). We further performed a Spearman correlation analysis among total, intact, 5′ defective, and 3′ defective proviruses (Figure 2E,F), revealing a distinct pattern of correlation across these four parameters in young and aged RMs. Similar to the human cohort study, we performed the IPDA on CD4− cell fractions from RMs to quantify the SIV myeloid reservoirs in PBMCs. Like PWH, RMs showed no significant differences in the levels of intact, 3′ defective, 5′ defective, and total proviruses of young versus aged RMs (Figure S3A). However, we observed a positive correlation between the intact versus total SIV proviral DNA copies of both young and aged macaques (Figure S3B,C). In summary, the non‐human primate studies corroborate the findings from human cohort studies, indicating significantly higher HIV reservoirs in the CD4+ T cells from the peripheral blood of older PWH. Notably, we did not observe significant differences in HIV/SIV reservoirs within the myeloid cells from PBMCs of young and old groups. While HIV reservoirs in lymphoid cells are primarily maintained through clonal proliferation of memory CD4+ T cells (Lee and Lichterfeld 2016), in the myeloid cell lineage, viral reservoirs predominantly persist in terminally differentiated, long‐lived tissue macrophages (Kumar et al. 2014). In a recent study, Veenhuis et al. (2023) comprehensively characterized replication‐competent HIV reservoirs in peripheral blood monocytes and monocyte‐derived macrophages (MDM) from ART‐suppressed PWH, using IPDA and QVOA (Veenhuis et al. 2023). Consistent with our findings, they showed that viral reservoirs in monocytes are stable over time, ranging from 9 months to 4 years, and proposed that these reservoirs, despite monocyte's short lifespan of approximately 72 h, may be continuously replenished by bone marrow derived hematopoietic progenitor cells (Carter et al. 2010; McNamara et al. 2013). A fraction of these monocytes may undergo diapedesis into tissues, differentiate into macrophages, and establish persistent viral reservoirs (Kruize and Kootstra 2019; Wong et al. 2019). The short lifespan and continuous replenishment of monocyte reservoirs from the bone marrow may explain why we do not observe an age‐related expansion in the size of HIV/SIV reservoirs in this PBMC‐derived CD4‐ cellular lineage.

One of the limitations of this study is the retrospective use of archived PBMC samples. Due to the limited number of cells available, we were only able to perform the IPDA and used previously generated flow cytometry data for correlation analysis. Unfortunately, this dataset lacked information on naïve and memory CD4+ and CD8+ T‐cell subsets. However, multiple studies have reported statistically significant reductions in naïve T‐cell populations, accompanied by an increase in memory T cells, in older individuals compared to younger counterparts in both HIV‐negative and PWH. These findings support our hypothesis that clonal expansion of memory T cells likely drives the increase in the size of HIV/SIV reservoirs in the older population (Chang et al. 2024; de Armas et al. 2021; Desai and Landay 2010; Heigele et al. 2015; M. Li et al. 2019; N. Li et al. 2025; Loste et al. 2024; Moro‐Garcia et al. 2013; Nikolich‐Zugich 2008; Provinciali et al. 2009; Rickabaugh et al. 2011; Saule et al. 2006). Moreover, several studies have shown that, in PWH on long‐term ART, viral reservoirs stop declining after approximately 7 years and instead begin expanding with an estimated doubling time of 23 years, indicating the proliferation of infected cells (McMyn et al. 2023). Additionally, due to the limited number of PBMCs available, we used the CD4+ T‐cell‐depleted (negative) fraction from PBMCs to estimate the myeloid reservoirs. This fraction contains all non‐CD4+ T cells, including a smaller proportion of myeloid cells, which are among the few non‐T‐cell types susceptible to HIV/SIV infection and thus serve as a reasonable proxy for assessing myeloid reservoirs. While the quantitative viral outgrowth assay (QVOA) is considered the gold standard for the measurement of replication‐competent HIV reservoirs, it provides only a minimal, yet definitive estimate of the frequency of cells carrying inducible, replication‐competent proviruses (McMyn et al. 2023). In contrast, the intact proviral DNA assay (IPDA) is high‐throughput, relatively inexpensive, requires fewer cells and is less time‐consuming, making it a practical alternative for reservoir estimation. Given the retrospective nature of our study and the limited availability of archived cells, we employed IPDA for quantifying the HIV/SIV reservoir, while acknowledging the respective strengths and limitations of both QVOA and IPDA.

In conclusion, this brief report suggests that the size of the HIV/SIV reservoirs in peripheral blood CD4+ T cells is higher in aged PWH/RMs as measured by IPDA. The interplay between HIV and aging involves complex immunological changes, and each contributes to chronic inflammation, factors that are likely to influence viral reservoir dynamics in secondary lymphoid organs like GALT, lymph nodes, and spleens, as well as within the CNS. Investigating these aspects is essential for devising effective strategies for curing HIV in the aging population. However, data from these anatomical sites remain limited due to the inaccessibility of human tissue specimens, particularly CNS tissues, during life. Therefore, the SIV‐infected rhesus macaque represents a preferred model to investigate these differences in tissues for a comprehensive understanding of the reservoir dynamics with aging.

## Author Contributions

Arpan Acharya and Siddappa N. Byrareddy conceived, designed, interpreted, and carried out the experiments. Debapriya Sutar contributed to experiments and data analysis. Suresh Pallikkuth and Varan Govind contributed to the human biological specimens and clinical data. Mahesh Mohan and Francois Villinger contributed rhesus macaque specimens and related data. Arpan Acharya and Siddappa Byrareddy drafted the manuscript. All authors critically reviewed and edited the final version of the manuscript.

## Conflicts of Interest

The authors declare no conflicts of interest.

## Supporting information


**Appendix S1:** acel70252‐sup‐0001‐AppendixS1.docx.

## Data Availability

All data presented in this manuscript are available in the main text or Supporting Information S1–S3.
