# Supplementary material for: Age‐Associated Expansion of HIV/SIV Reservoirs in People With HIV and SIV‐Infected Macaques
Source: Aging Cell. 2025 Oct 6;24(12):e70252. doi: 10.1111/acel.70252 (PMC12686580; doi:10.1111/acel.70252)
Supplement: Supplementary file 1 — Appendix S1: acel70252‐sup‐0001‐AppendixS1.docx. [file ACEL-24-e70252-s001.docx]

**Supplementary Table 1:**

| **Demographic and clinical characteristics of the study cohort** | | | |
| --- | --- | --- | --- |
| **Characteristics** | **Young (n=15)** | **Old (n=14)** | **p Value** |
| Age at study entry (years; mean±SD) | 28.73±6.82 | 64.57±5.00 | <0.0001 |
| Age range (years) | 19-39 | 60-77 |  |
| Sex, n (%) | | | |
| Male | 10 (66.67) | 8 (57.15) |  |
| Female | 5 (33.33) | 6 (42.85) |  |
| Ethnicity, n (%) | | | |
| Non-Hispanic | 9 (60.00) | 9 (64.29) |  |
| Hispanic | 6 (40.00) | 5 (35.71) |  |
| Race, n (%) | | | |
| White | 7 (46.67) | 5 (35.71) |  |
| Black | 8 (53.33) | 9 (64.29) |  |
| Years on ART at sampling | 5.2 ±2.5 | 16.9 ± 4.6 | <0.01 |
| Plasma Viral Load at sampling (copies/ml) | <50 | <50 |  |
| SD: Standard deviation; ART: Antiretroviral therapy | | | |

**Supplementary Table 2: Rhesus macaque study cohort details**

| **Animal ID** | **Age Group** | **Study Group** | **Sex** | **Age at study entry** | **ART started (days post-infection)** | **No. of days on ART at time of sample collection** |
| --- | --- | --- | --- | --- | --- | --- |
| A13T016 | Young | Control | Male | 4 | 84 | 948 |
| A13T022 | Young | Control | Male | 4 | 84 | 948 |
| 14R452 | Young | Control | Male | 5 | 84 | 336 |
| A15N080 | Young | Control | Male | 4 | 84 | 336 |
| A17X003 | Young | Control | Male | 2 | 84 | 294 |
| A17X013 | Young | Control | Male | 2 | 84 | 336 |
| 97N080 | Old | Control | Female | 21 | 84 | 635 |
| 99N018 | Old | Control | Female | 19 | 84 | 635 |
| 99N088 | Old | Control | Female | 19 | 84 | 635 |
| 43976 | Old | Control | Male | 22 | 14 | 150 |
| 43978 | Old | Control | Male | 19 | 14 | 210 |
| 43977 | Old | THC/CBD | Male | 21 | 14 | 210 |
| 43979 | Old | THC/CBD | Male | 19 | 14 | 150 |
| 43980 | Old | THC/CBD | Male | 19 | 14 | 210 |

**
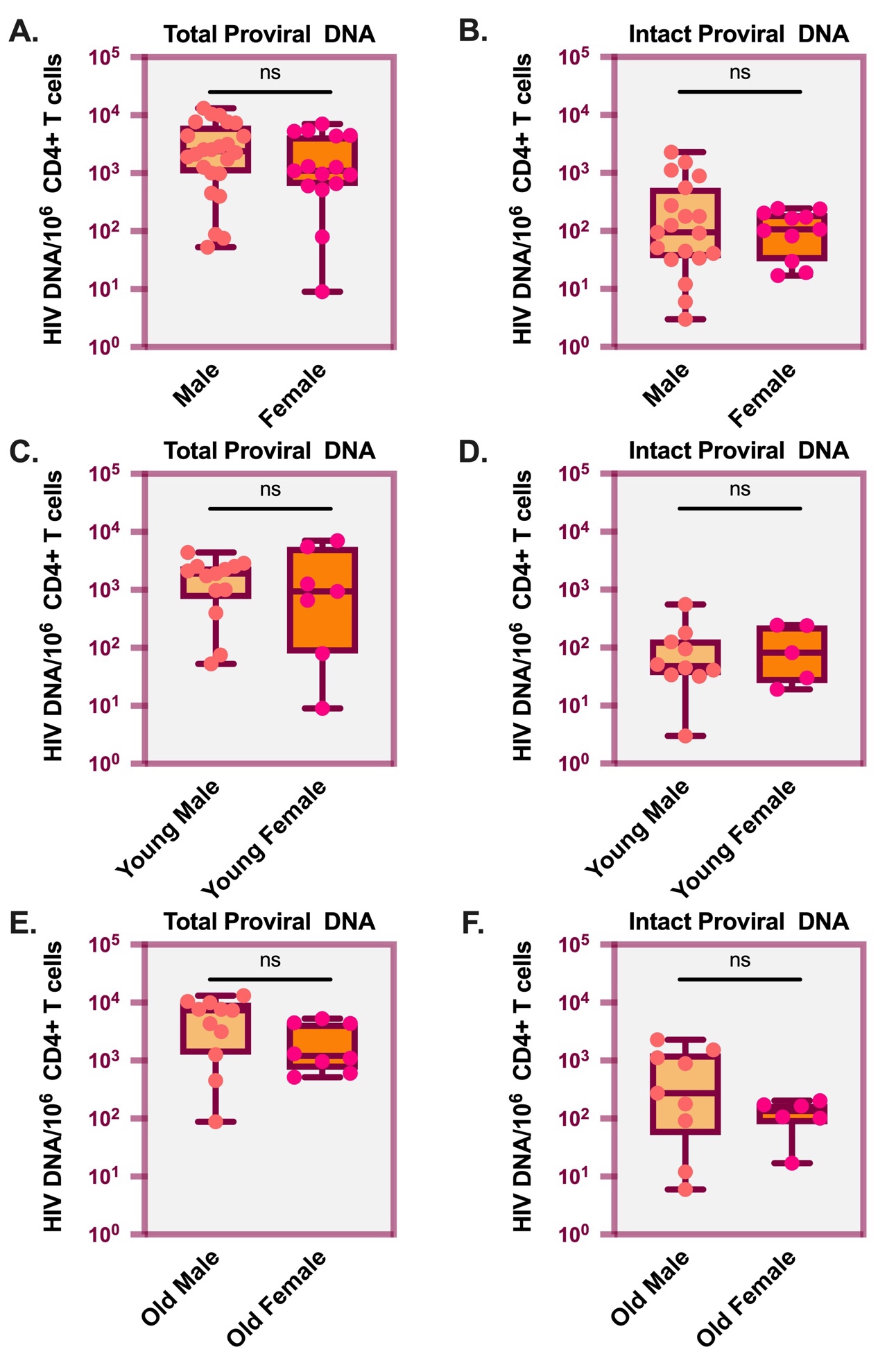
**

**Supplementary Figure 1. Comparison of HIV reservoir levels in CD4+ cells from peripheral blood between male and female people living with HIV (PWH). (A)** Frequency of total proviral DNA per million CD4+ T cells purified from PBMCs between male versus female PWH (includes young and old study cohort); **(B)** Frequency of intact proviral DNA per million CD4+ T cells purified from PBMCs between male versus female PWH (includes young and old study cohort); **(C)** Frequency of total proviral DNA per million CD4+ T cells purified from PBMCs between young male versus female PWH; **(D)** Frequency of intact proviral DNA per million CD4+ T cells purified from PBMCs between young male versus female PWH; **(E)** Frequency of total proviral DNA per million CD4+ T cells purified from PBMCs between old male versus female PWH; **(F)** Frequency of intact proviral DNA per million CD4+ T cells purified from PBMC between old male versus female PWH; ns: not significant.

**
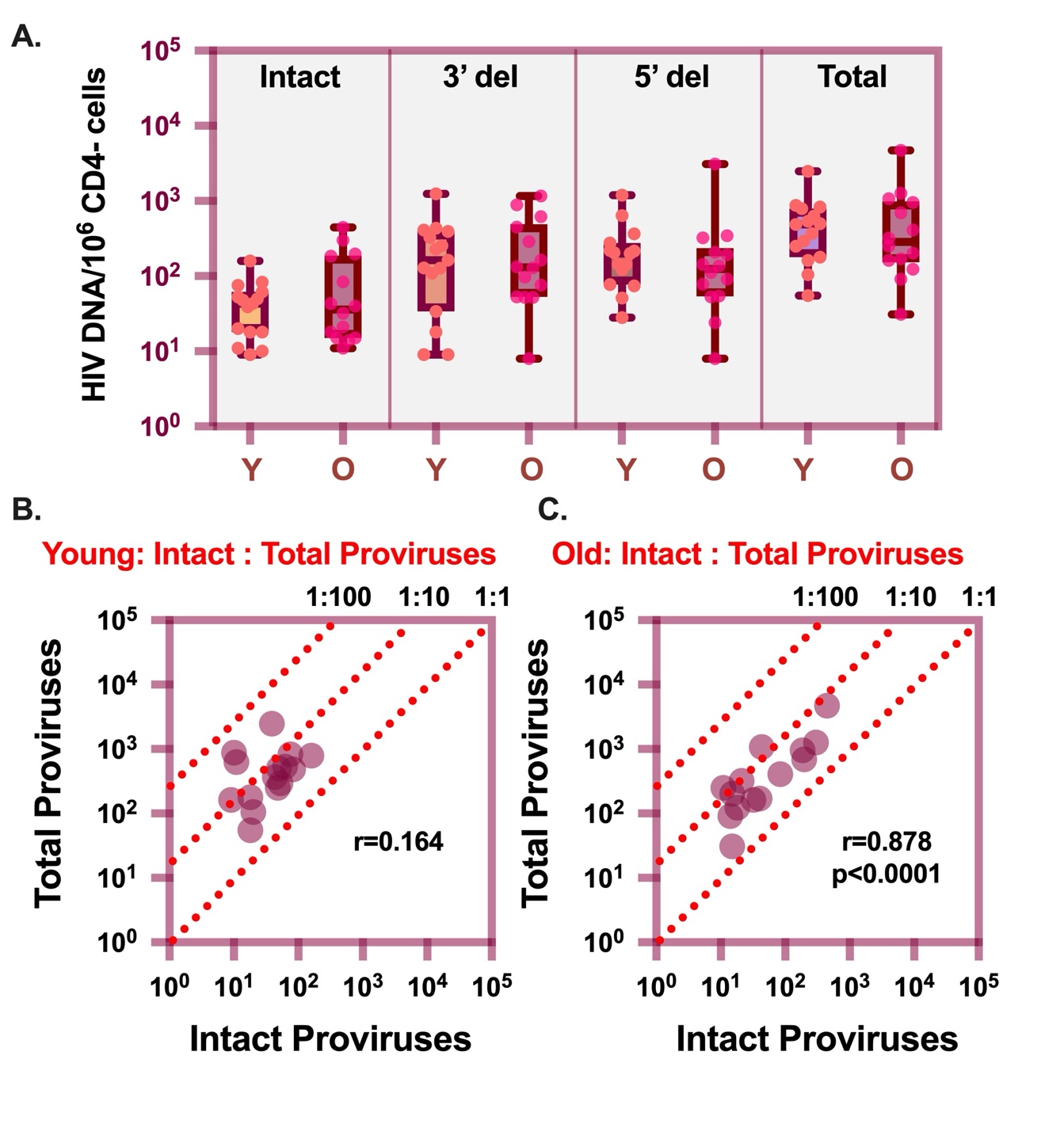
**

**Supplementary Figure 2. Comparison of HIV reservoir levels in CD4- fractions from peripheral blood between young and old people with HIV (PWH). (A)** Comparison of intact, 3' deleted, 5' deleted, and total proviral DNA per million cells between young versus old PWH; **(B)** Spearman correlations plot between intact and total HIV proviral DNA levels of young PWH; **(D)** Spearman correlations plot between intact and total HIV proviral DNA levels of old PWH.

**
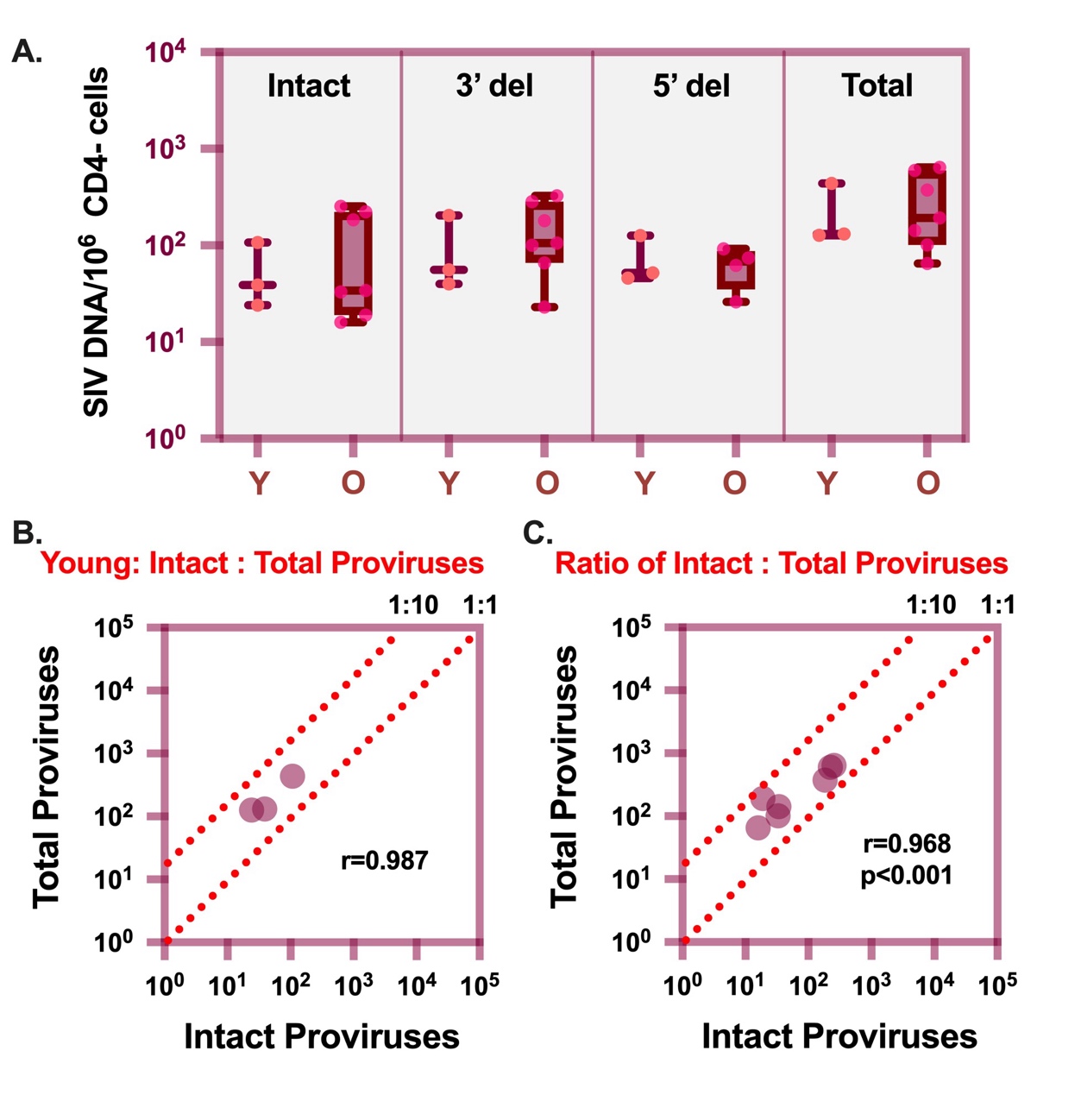
**

**Supplementary Figure 3. Comparison of SIV reservoir levels in CD4- fractions from peripheral blood between young and old rhesus macaques. (A)** Comparison of intact, 3' deleted, 5' deleted, and total proviral DNA per million cells between young versus old SIV-infected ART-treated rhesus macaques; **(B)** Spearman correlations plot between intact and total SIV proviral DNA levels of young SIV-infected ART-treated rhesus macaques; **(C)** Spearman correlations plot between intact and total SIV proviral DNA levels of old SIV-infected ART-treated rhesus macaques; The dashed lines correspond to relative ratios of intact to total proviral DNA.
